# Self-Healing Performance Evaluation of Concrete Incorporating Inorganic Materials Based on a Water Permeability Test

**DOI:** 10.3390/ma14123202

**Published:** 2021-06-10

**Authors:** Kwang-Myong Lee, Hyung-Suk Kim, Do-Keun Lee, Kyung-Joon Shin

**Affiliations:** 1 Department of Civil, Architectural and Environmental System Engineering, Sungkyunkwan University, Suwon 16419, Korea; leekm79@skku.edu (K.-M.L.); kimhs775@skku.edu (H.-S.K.); 2 Department of Civil Engineering, Chungnam National University, Daejeon 34134, Korea; likedg@o.cnu.ac.kr

**Keywords:** self-healing, performance evaluation, self-healing additives, water permeability test

## Abstract

Research activities that have focused on the development and understanding of self-healing concrete have proposed various technologies intended to enhance self-healing capacity. The self-healing performance cannot be identified sufficiently with either a single test or a specific parameter because there are a number of factors that influence the performance of self-healing. Thus, it has become necessary to provide standardized test methods that make it possible to verify and compare the performance of self-healing materials. In this paper, self-healing mortars based on inorganic admixtures, which are developed for sealing 0.3 mm cracks with a healing index of 90%, are produced and used to validate the water permeability test and to propose protocols for the evaluation of self-healing performance. The healing performances of three self-healing mortars and a plain mortar as a reference are evaluated with a comparative study. The equivalent crack width, which can be estimated from the water flow rate, is suggested as a rational evaluation index. Finally, a self-healing performance chart is proposed to comprehensively show the healing performance of cement-based materials.

## 1. Introduction

Concrete is a widely used construction material because it possesses several beneficial features, such as its high compressive strength, economic feasibility, and tractability. However, one of the primary disadvantages of concrete is that cracks frequently occur. These cracks can result from several causes, including an excessive load applied on structures, a volume change accompanying the hardening process, and inherent cracks due to the nature of reinforced concrete structures. These cracks deteriorate the durability and serviceability of concrete structures. Thus, both the control and prevention of cracks are required for concrete.

As the importance of life-cycle maintenance and the elongation of the service life of infrastructure are becoming increasingly important for their sustainable use all over the world, many studies have focused on improving the efficiency of the maintenance of structures. However, with concrete in particular, it is not easy for repairs and the rehabilitation of cracks in structures to be promptly undertaken. To address the problem of cracks in concrete, self-healing technologies, which allow cracks to be repaired and controlled by themselves, have been actively studied in recent years [1,2,3]. 

Various materials such as mineral admixtures, geomaterials, bacteria, and microcapsules are gradually being explored for use as self-healing materials [1,2,3,4,5,6,7]. Each of these materials has its own characteristics that contribute to self-healing. To evaluate and compare the self-healing performance of such self-healing materials, conventional test methods have been used, including permeability tests, microstructural analysis, image analysis, mechanical tests, non-destructive tests, and ionic diffusion [3,8]. Research to develop a test method suitable for self-healing concrete with cracks is also of interest [9,10,11,12]. However, because studies in the field of self-healing technology for concrete structures have only been actively conducted over the last two decades, standardization of evaluation methods and protocols for self-healing performance have not yet been established along with criteria and indexes, although several have been proposed [13,14,15].

For the evaluation of self-sealing performance related to durability, a water permeability test has been used the most. The water permeability test can evaluate the performance of self-healing by measuring the amount of water passing through a crack. As self-healing concrete research has been actively pursued, various types of water permeability tests have been used [1,3,8,11,12,13,14,15,16,17,18,19,20]. However, because no standardized method is available for the permeability test, there is a large variety of permeability test results, even for specimens with similar crack widths. 

Autogenous healing is one of the major mechanisms for self-healing concrete. It is a well-known phenomenon that originates from natural processes in cementitious material, such as the hydration of clinker minerals or the carbonation of calcium hydroxide, and it is considered to be the most popular approach for practical applications. A recent study showed that autogenous healing could be enhanced using mineral admixtures such as ground-granulated blast-furnace slag (GGBFS) and crystalline admixtures [21,22]. Another study investigated the proper distribution of cement particle sizes that could provide a suitable amount of Ca(OH)_2_ and unhydrated cement and thereby improve the self-healing ability of concrete [23]. As such, various methods have been applied for the enhancement of healing. However, to date a thorough direct comparative evaluation of these methods has not been reported. Accordingly, this study examines the autogenous healing of mortar containing various types of healing agents.

Therefore, this study proposes a method and protocol for the evaluation of self-healing performance based on a water permeability test. The performance of three self-healing mixtures incorporating inorganic materials have been investigated and compared for the verification of the theory, test method, and analysis methods. Three self-healing mixtures were used. The first mixture (SH1) used capsules containing mineral admixtures. The second mixture (SH2) utilized a combination of admixtures to promote expansion, swelling, and crystal growth. The third mixture (SH3) used a cement clinker as an additive. A series of tests for cracked mortar specimens were conducted to verify the proposed method. Following this, the analysis methods were discussed for the interpretation of the experimental results. 

## 2. Self-Healing Performance Evaluation

### 2.1. Self-Healing Performance Evaluation Based on a Water Permeability Test

Current self-healing technology aims to recover the water tightness of cracks or the mechanical performance in concrete, and appropriate performance evaluation methods have been used [3]. For performance evaluation related to durability, the water permeability test has been used the most because of the relative ease of the test, which can be performed without complicated equipment [11,17,18,19]. 

As self-healing concrete research has been actively pursued, the water permeability test is receiving more attention. Although it has been primarily used as an evaluation tool, the detailed experimental procedures have been different for each researcher [3,14,15,16,17,18]. As no standardized method is available for the permeability test, there are large variations in the permeability test results, even for specimens with similar crack widths. There are several reasons for this large variation in results. It has been theoretically proven that some parameters related to the test setup influence the results [18]. In addition, in our experience, the permeability test can be influenced by minor parameters that are theoretically not supposed to affect the results, such as the water head, the thickness of the specimen, and the size of drainage pipe. Therefore, we have proposed a water permeability test method with refined details for the apparatus and test conditions. We also present a protocol for the self-healing performance evaluation of concrete that is based on a constant water head permeability method using a cylindrical cracked specimen.

### 2.2. Correlation between Crack Width and Water Flow Rate of Cracked Mortar Specimens

In general, water flow passing through a crack can be idealized as a flow between two plates. The water flow rate Q through a crack can be derived by Poiseuille’s law [16,24]:

(1) $$Q=\xi \frac{\Delta Pb}{12\eta d}w^{3}$$

where, Δ*P* is the water head gradient between the inlet and the outlet of the crack, *b* is the length of the crack, w is the crack width, η is the absolute viscosity, *d* is the flow path length of the crack, and ξ is a reduction factor reflecting the roughness of the crack. 

In Equation (1), when a constant water head is applied, it is assumed that Δ*P* and η are constant and d is equal to the thickness of the specimen. With these assumptions, the water flow per unit length of a crack, q, is given by:

(2) $$q=\frac{Q}{b}=\xi \frac{\Delta P}{12\eta d}w^{3}=\alpha w^{3}$$

where α is a coefficient of proportionality that relates the water flow rate to the third power of the crack width.

If α is determined, the water flow rate through a crack can be predicted with Equation (2) once *w* is measured. Conversely, the initial crack width (${\bar{w}}_{0}$, the overline markup means estimated, not measured) can be assessed using a measured initial water flow rate ($q_{0})$, as follows.

(3) $${\bar{w}}_{0}={\left(q_{0}/\alpha \right)}^{\frac{1}{3}}$$

**Figure 1:**
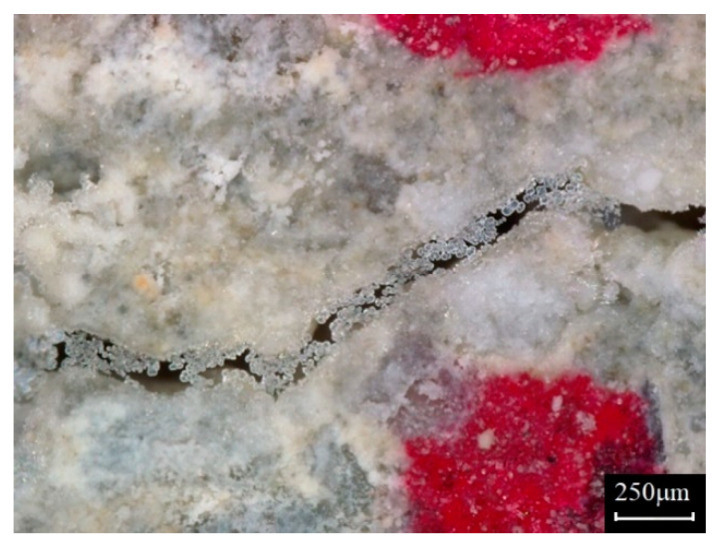
Irregular healing products on the crack surface.

Therefore, in cases where the crack width is difficult to measure or the measurements are not reliable, the crack width can be theoretically evaluated using the water permeability test results. For example, in the middle of the healing process, assessing the crack width is almost impossible due to the irregular healing products on the inner surfaces of specimens, as shown in Figure 1. In this case, the crack width can be estimated using the pre-determined coefficient *α* with:

(4) $$\bar{w}\left(t\right)={\left(q\left(t\right)/\alpha \right)}^{\frac{1}{3}}$$

where, $\bar{w}\left(t\right)$ is the estimated crack width at time *t*, *q*(*t*) is the water flow rate at t, and t is the healing period. The crack width derived in this way is defined as *the equivalent crack width*, as it is not definite but rather a hypothetical value that is indirectly predicted from the water flow rate. The relationship between the crack width and the flow rate could change because there is a possible property change of the crack surface due to the healing process. However, assuming that there is little change in the crack width–flow rate relationship due to healing, it is possible to estimate the change in the equivalent crack width throughout the healing processes. Even though the equivalent crack width is not the actual crack width, it can be a reasonable basis for evaluating the self-healing performance. 

### 2.3. Evaluation of Self-Healing Performance Using the Water Permeability Test

As self-healing progresses, the healing products fill the cracks. As the crack width decreases, the water flow rate also decreases. Generally, the self-healing performance of concrete can be evaluated using the water flow reduction ratio, as in [3,13,25]:

(5) $$SH_{q}=\left[1-\frac{q\left(t\right)}{q_{0}}\right]\times 100\;(\%)$$

where, $SH_{q}$ is the self-healing index based on the water flow rate and $q_{0}$ is the initial water flow rate measured just after the specimen is cracked without any healing effect. The derived value of *SH_q_* for *q*(*t*) can be interpreted as how much of the water tightness is recovered.

## 3. Experimental Works

### 3.1. Test Plan

The three developed mixtures as well as a plain mortar mixture, used as a reference and referred to as Plain, were used to demonstrate the enhanced healing performance of the specially designed self-healing additives. Using these mortars, the proposed test method and protocol were verified. The experimental works consisted of two steps. The first step validated the proposed method for estimating the crack width of specimens based on the water flow rate. A total of 160 specimens were made (34~46 specimens for each mixture). A single crack was induced 28 days after casting. The crack widths of the specimens ranged from 70 to 320 µm. The correlation between the crack width and the water flow rate was confirmed using these cracked specimens. The second step evaluated the self-healing performance of the mortar mixtures containing the different self-healing enhancing additives. To clearly compare the healing effects between specimens, specimens with crack widths of 200~300 µm were selected and healed for 28 days. A total of 40 specimens (10 specimens × 4 mortar mixtures) were chosen among the specimens used in the first step. 

### 3.2. Materials and Mixture Proportions

Four different mixtures were used to compare and analyze the performance of the self-healing mortar mixtures that were developed for sealing a 0.3 mm crack up to an *SH_q_* of 90%. One was the Plain reference mixture. The other mixtures contained the self-healing admixtures, which have their own unique characteristics. For all of the mixtures, Type I portland cement produced in Korea (Ssangyong Cement, Yeongwol) was used as a binder. ISO standard sand with a density of 2.60 g/cm^3^ and a fineness modulus of 2.95 was used as the fine aggregates. Table 1 shows the mix proportions of the mortar mixtures. A water/cement ratio (W/C) of 0.4 and a cement/sand ratio (S/C) of 2.0 was used for all mixtures. 

Mixtures SH1 and SH2 replaced 2.5 or 4% of the fine aggregates with healing agents, respectively. The SH1 mixture adapted a capsuling technique to enable the healing agent to act effectively. Expansive admixtures and anhydrate gypsum were used for the healing agent. The agent was encapsulated using a urethane-based coagulant and a liquid rubber coating. The SH2 mixture combined a variety of components such as expansive agents, geomaterials, and materials that promote crystal growth. The healing agent consisted of sodium carbonate (Na_2_CO_3_) and zeolite embedded with calcium stearate. The relative proportions of Na_2_CO_3_, calcium stearate, and zeolite were 1:0.5:1.5. To achieve proper mixing, Na_2_CO_3_ was dissolved in water, then the zeolite was added to the mixture. The mixture was dried at 40 °C for 24 h and immediately dried at 100 °C for 72 h. Then, the healing agent was obtained by grinding these dried materials with calcium sulfoaluminate (CSA). The SH3 mixture used clinker particles with an average diameter of 2.5 mm to replace 10% of the fine aggregates and clinker particles with an average diameter of 0.85 mm to replace 15% of the cement.

### 3.3. Preparation of Test Specimens

**Figure 2:**
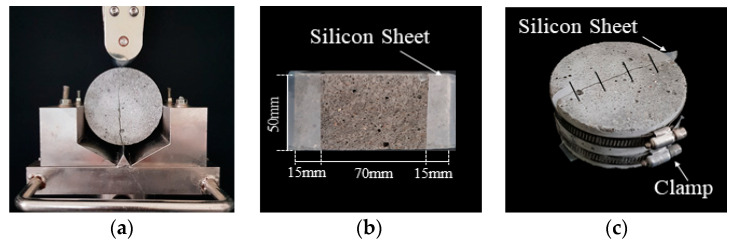
Preparation of cracked specimens: (**a**) split specimen, (**b**) silicon rubber sheets located between semicircular sections, and (**c**) specimen bound together with steel bands.

The cracked specimens were produced through several steps. First, the mortar cylinders (Ø100 mm × 200 mm) were prepared. Next, these cylinders were demolded after 24 h and were cured in a water bath at 20 °C until they reached the crack-inducing age of 28 days. Once the cracking age was attained, the cylinders were sliced into a disc shape (Ø100 mm × 50 mm) and then split into two semicircular sections, as shown in Figure 2a. Then, a flexible silicone rubber sheet was attached to both ends of the cracked sections to induce a crack of specified width, as shown in Figure 2b. The desired crack widths were achieved using silicone rubber sheets with varying thicknesses. It should be noted that these silicon sheets could control the crack widths and prevent lateral leakage due to their flexible characteristics. The actual lengths of the cracks were approximately 70 mm. Finally, the split specimens were bound together using stainless steel bands to maintain the desired crack widths, as shown in Figure 2c. The crack width could be controlled finely by adjusting the tightness of the steel band. This proposed method has the drawback that the specimen needs to be split such that the original crack surface can be disturbed. However, the method has the strong advantage that it can control the crack width and prevent leakage with minimum effort by simply placing the silicon sheets between a crack and tightening the specimen with a steel band.

**Figure 3:**
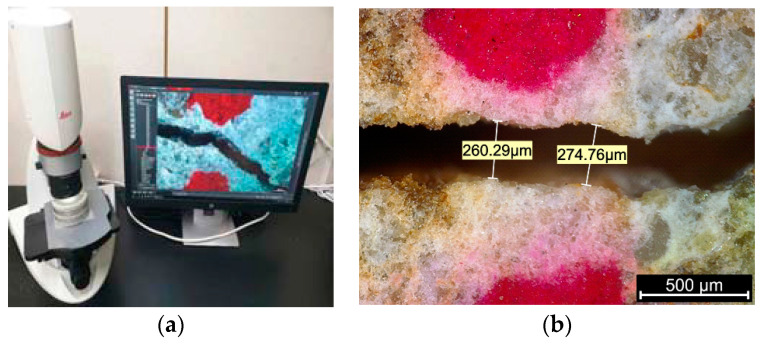
Measurement of crack width: (**a**) microscope measurement system, (**b**) crack width.

After the specimens were prepared, the widths and lengths of cracks were measured using a microscope (Leica Microsystems, Wetzlar, Germany), as shown in Figure 3. Crack widths were measured at a total of 12 locations on the top and bottom surfaces. Then the average value was recorded as the crack width of the specimen. The COVs (coefficient of variation) of the measured crack widths for these specimens were 3.7~13.5%. Crack lengths were also measured at both the top and bottom surfaces.

### 3.4. Water Permeability Test Method

In general, with soil, two kinds of water permeability methods are applied depending on the amount of outflow. For a large amount of outflow, a constant water head permeability test method is usually applied, while with a small amount of outflow a variable water head permeability test is commonly used. For testing a cracked concrete specimen, each of these methods have been used [1,11,16,26]. In this study, a constant water head permeability test method was applied for the following reason. When the permeability test is used for the performance evaluation of self-healing, the water flow rate is supposed to vary with time. Typically cracked specimens initially exhibit a large amount of outflow, but the outflow gradually decreases because the cracks are filled with healing products as the age increases. The water head is one of the major parameters influencing the permeability. A loss of water head is supposed to occur during the permeability test [21]. This head loss is known to increase with an increase in water velocity and is difficult to estimate or compensate for theoretically [18]. Therefore, to minimize the variation of the unexpected head loss that occurs during the test and to keep the test condition uniform, a constant water head method was adopted.

**Figure 4:**
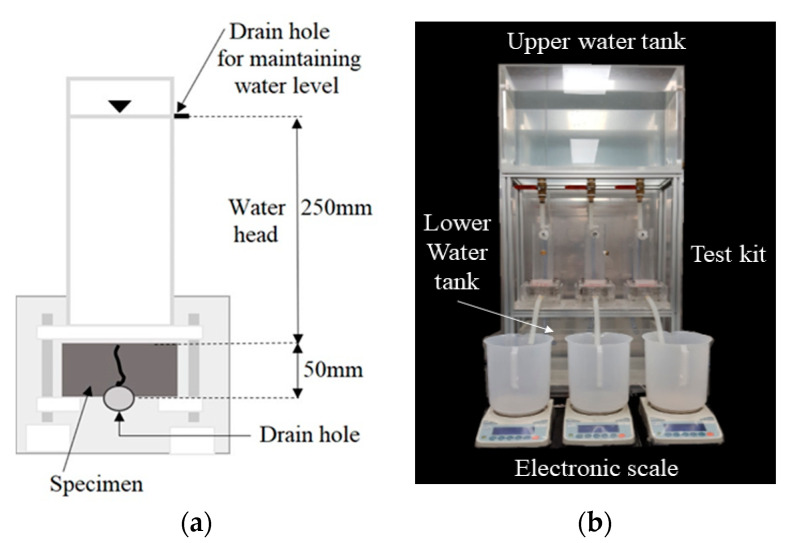
Water permeability test apparatus: (**a**) schematic diagram, (**b**) photograph.

Figure 4 shows the equipment for the water permeability test for the cracked specimen. It should be noted that a constant water head of 300 mm was maintained in this study. In addition, the size of the drainpipe should be large enough not to influence the overall head loss. If the area of the outlet is 10 times larger than the crack area, the velocity in the outlet becomes less than 10% of the velocity in the crack, allowing minor losses in the outlet hose to be ignored [18].

The amount of water coming out of the test equipment was measured for 7 min after the water head and water flow stabilized. To normalize the results for specimens with various crack shapes, the test duration and crack length were considered. The water flow rate in units of mL/(mm·min) was obtained by dividing this amount of discharged water by the test duration (min) and crack length (mm). 

The cracked specimens were cured separately in water at 20 °C and grouped by the mixture type to prevent possible ion-exchange that could occur during the healing period. The amount of water was also controlled to be 500 mL for each specimen. This was achieved by adding the evaporated amount of water back into the containers once per week after the water permeability testing. With this process the possible effect of curing water was minimized.

The water permeability test was conducted for healing periods of 0, 7, 14, 21, and 28 days. The specimens were stored in the same container between subsequent measurements. Because there was the possibility of disturbing the self-healed products of the specimens, preconditioning, such as vacuum saturation, was not performed before the permeability test. 

## 4. Experimental Results and Discussion

### 4.1. Fundamental Properties

Fundamental properties such as the compressive strength and the slump flow were measured. The compressive strengths of the mortars were measured according to KS L ISO 679 “Methods of testing cements-Determination of strength.” Table 2 shows the strength measured at 7 and 28 days after curing. The 28-day strengths of self-healing mixtures (SH1, SH2, SH3) were 106.6%, 92.3%, and 108.1% of that of the Plain mixture. 

The slump flow was measured according to ASTM C 1437 methods. As shown in Table 2, the initial slump flows of the self-healing mixtures were similar to that of the Plain mixture. The initial slump flows of the self-healing mixtures were 100%, 108.3%, and 97.2% of that of the Plain mixture, respectively. However, the SH3 mixtures showed a little more loss of flow compared to that of the others after 60 min. 

### 4.2. Correlation between Crack Width and Water Flow Rate

**Figure 5:**
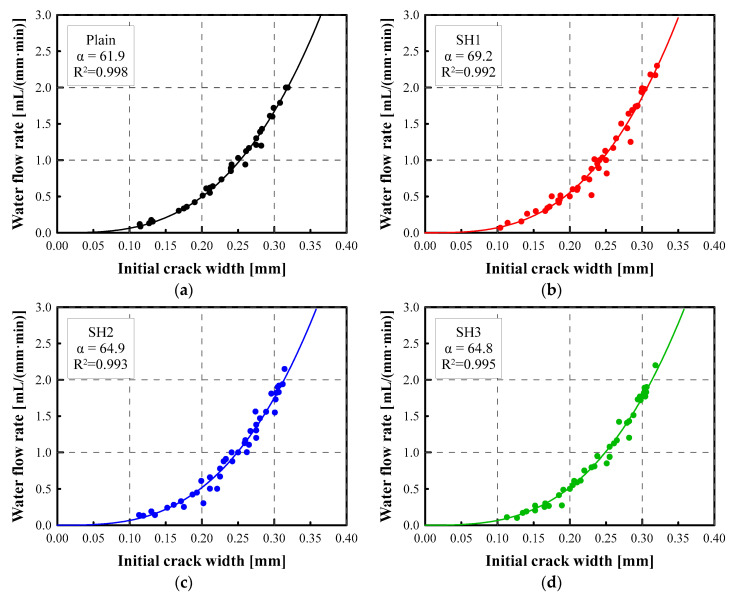
Relationship of crack width and water flow rate: (**a**) Plain, (**b**) SH1, (**c**) SH2, (**d**) SH3.

In this study, to demonstrate a correlation between the crack width and the water flow rate, extensive tests were conducted with 160 specimens using the proposed apparatus and protocols in a controlled environment. Figure 5 shows the relationship between the initial water flow rate (mL/(mm·min)) and the crack width (µm) of the tested specimens for the Plain, SH1, SH2, and SH3 mixtures. The specimens were cracked after 28 days of curing. The initial flow rate increased with respect to the cube of the crack width. These relationships can be modeled well using Equation (2). The proportional coefficient α was determined for each mixture, as shown in Figure 5. The coefficients of determination (R^2^) calculated during the parameter estimation were larger than 0.99. This implies that the theoretical equations explained in Section 2 are valid for the test results of the specimens with the same mixture. 

According to Poiseuille’s law, the reduction factor (ξ) in Equation (1) should be 1.0 for an ideal crack. However, due to the roughness and tortuosity of the cracks, the proportional coefficients measured are in the range of 61.9~69.2 mL/(mm·min). This corresponds to a ξ of 0.21~0.24. This range is similar to the values of 0.22~0.25 for mortar mixtures with W/C = 0.48 and S/C = 0.8 [27] and also similar to the value of 0.25 from the experimental results obtained with concrete mixtures [16]. 

It should be noted that the type of mixtures and the age of the cracked concrete determines the relationship between the water flow rate and the crack width, which can be represented with the proportional coefficient α. This is due to the fact that the flow rate in the crack is primarily affected by the roughness of the crack surface. The characteristics of the crack surface are mainly affected by the mixture proportions and the curing ages [28]. 

Using the relationship between the crack width and the water flow rate given in Equation (3), the crack width can be estimated from the water flow rate. Table 3 lists the microscope-measured crack widths alongside the crack widths estimated from the flow rates. Just after the specimens were cracked, the crack widths were measured, and the permeability test was conducted without any self-healing phenomena. According to the table, the crack widths estimated from the initial flow rate are within 2.37%, or 6 µm, of the measured initial crack widths. 

The initial flow rate can also be estimated from the measured crack widths through Equation (2). However, the estimated flow rate had an error of up to 14.9% compared to the measured initial flow rate, because the water flow rate is proportional to the cube of the crack width so that the error in the estimated flow rate is much larger than that in the estimated crack width.

### 4.3. Evaluation of Self-Healing Performance

#### 4.3.1. Optical Observation of the Healing Phenomena 

**Figure 6:**
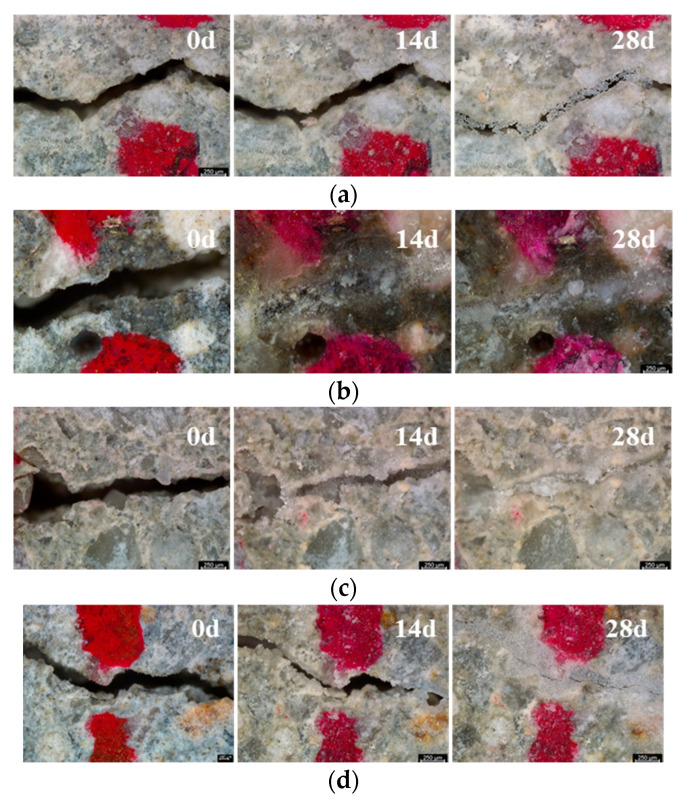
Surface cracks for healing periods 0, 14, and 28 days: (**a**) Plain, (**b**) SH1, (**c**) SH2, (**d**) SH3.

After cracking, if the specimen is exposed to the appropriate environment, the crack can be sealed by the self-healing phenomena, leading to a reduction in the crack width. The self-healing phenomena can be observed with an optical microscope. Figure 6 shows the healing processes of the test specimens. It seems that the cracks are almost filled with the healing products for SH1, SH2, and SH3 specimens while there still is a gap in the Plain specimens. When comparing the 14-day specimens, it can be seen that the SH1 mixtures have a better healing performance than the others. 

Because an optical observation of the cracks is limited to the surface of the specimen, a simple surface observation cannot be representative of the whole crack. In addition, the degree of the surface crack filled cannot be representative of the healing of the whole depth of the crack. Thus, this self-healing process is generally estimated from the evaluation of other properties, such as water permeability, chloride permeability, or flexural strength [11,15,16,17,18]. A computed tomography (CT) based on 3D scanning can effectively capture the information of the filled crack [29]. However, due to the limitation of the availability of the equipment, it is difficult for it to be a commonly used test method. Thus, as addressed in Section 4.1, a permeability test can be an effective method for measuring the change in the crack width. 

#### 4.3.2. Measurement of Water Permeability

**Figure 7:**
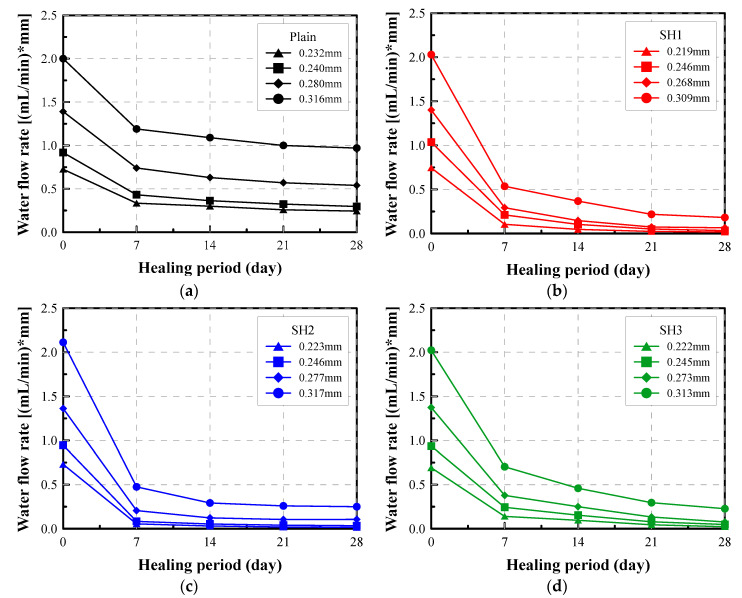
Water flow rate vs. healing period of selected specimens: (**a**) Plain, (**b**) SH1, (**c**) SH2, (**d**). SH3.

To quantify the healing of the crack, water flow rates were measured using the constant water head permeability test. Figure 7 shows the typical patterns for the variation of the water flow rate of selected self-healed specimens during the test period. The test results show that the water flow rates decreased with the increase in the healing period, regardless of the mixture types and initial crack widths. However, the rate of decrease varied depending on the type of mixtures. This reveals that the healing products seal cracks via a further hydration of the cement and other self-healing mechanisms, including blockage by impurities or shifts in the products, and this leads to the reduction in the water flow rate. Overall, more than half of the healing occurs within 7 days, but the figures show that the detailed characteristics of healing varied. 

Figure 7a shows that the flow rates of the Plain mixture were not reduced much compared to those of the self-healing mixtures. While the flow rates of the Plain mixture remained above 0.2 mL/(mm·min) after 28 days, even for the specimens with a narrow crack width, the flow rate of the self-healing mixtures decreased down to below 0.2 mL/(mm·min), even for the specimens with a relatively wide crack. 

The trend for the flow rate to decrease, which reflects the healing characteristics, varied for each specimen. Specimens with initial water flow rates of 1.5 mL/(mm·min) or more continued to show a large flow rate even after 28 days. However, the flow rate of the specimens with a relatively low initial flow rate was reduced to around 0.5 mL/(mm·min) for the Plain mixture or to 0.1 mL/(mm·min) for the self-healing mixtures. 

Figure 7b–d proves that the self-healing effect can be clearly shown by the reduction in the water flow. However, it is not easy to intuitively understand the meaning of the value of the flow rate and to figure out how much the crack is sealed. To correct this, the healing index and the equivalent crack width can be adopted as an effective index for healing.

#### 4.3.3. Relative Change in Permeability: Healing Index

**Figure 8:**
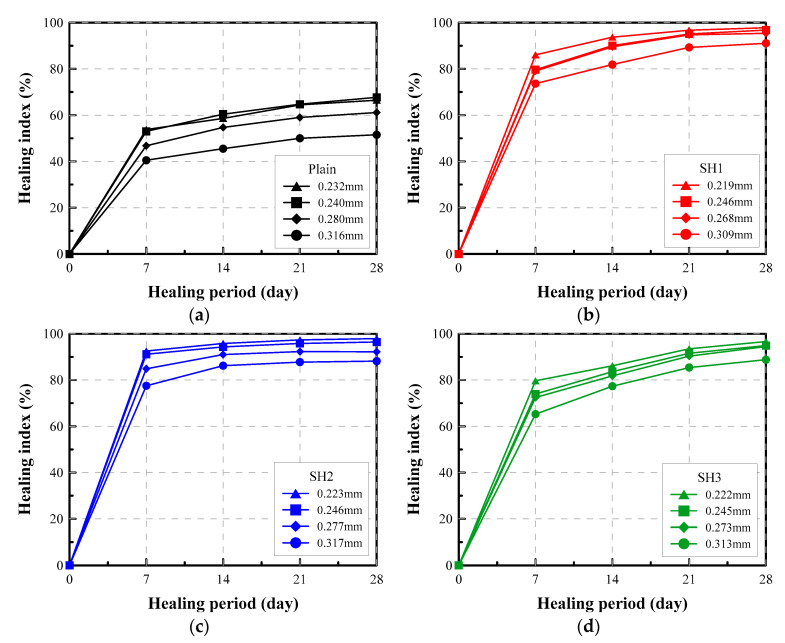
Healing index vs. healing period of selected specimens: (**a**) Plain, (**b**) SH1, (**c**) SH2, (**d**) SH3.

The self-healing performance can be evaluated by calculating the healing index, which is expressed as a relative change of the permeability, by using Equation (5). This healing index can be interpreted as the reduction rate of water flow attained at a given period. Figure 8 shows the variation of the healing index for different mixtures with various crack widths during 28 days of healing. The healing indices of the Plain specimens increased from about 50% up to 65% when the initial crack width decreased from 0.32 to 0.23 mm. However, the healing indices of the SH1, SH2, and SH3 specimens increased above 90% when the initial crack widths were less than 0.30 mm. This means that the water flow passing through a crack of less than 0.3 mm can be reduced by more than 90% by using the self-healing mixtures. Among the mixtures, the SH2 mixtures showed the fastest healing process. The SH2 mixture could attain more than an 80% healing index for a crack of less than 0.3 mm over a healing period of 7 days. 

**Figure 9:**
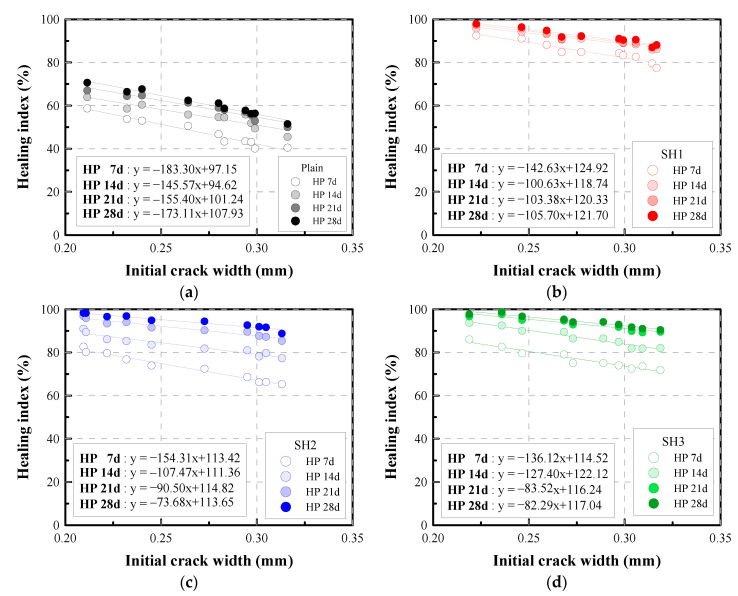
Relationship between the self-healing index and the initial crack width: (**a**) Plain, (**b**) SH1, (**c**) SH2, (**d**) SH3.

For further investigation, the relationships between the healing index and the initial crack width are shown in Figure 9. It shows the healing index at a certain time is highly related to the initial crack width. The correlation coefficients between the crack width and the healing index are calculated to be at least 0.92 and on average 0.96, so that the relationship can be expressed as a linear equation through the regression analysis, as shown in Figure 9. The equation clearly shows the potential self-healing performance of the mixtures. For example, if the expected initial crack was 0.25 mm, then the expected healing performance of the Plain, SH1, SH2, and SH3 mixtures after 28 days would be 0.65, 0.95, 0.95, and 0.96, respectively. Alternatively, if the targeted healing index is 0.90 at 28 days, then the maximum allowable initial crack width that can be healed would be calculated to be 0.10, 0.30, 0.32, and 0.33 mm for the Plain, SH1, SH2, and SH3 mixtures, respectively. In this way, this equation could be used before the use of self-healing materials to confirm the expected possible healing capacity.

#### 4.3.4. Equivalent Crack Width 

The healing index could be an effective way to indicate how much the crack is sealed. However, it is not easy to correlate intuitively the healing index to the crack width because the healing index is a relative concept. To compensate for this, the equivalent crack width proposed in Section 2.2 can be adopted as an effective index to indicate an absolute degree of healing. 

**Figure 10:**
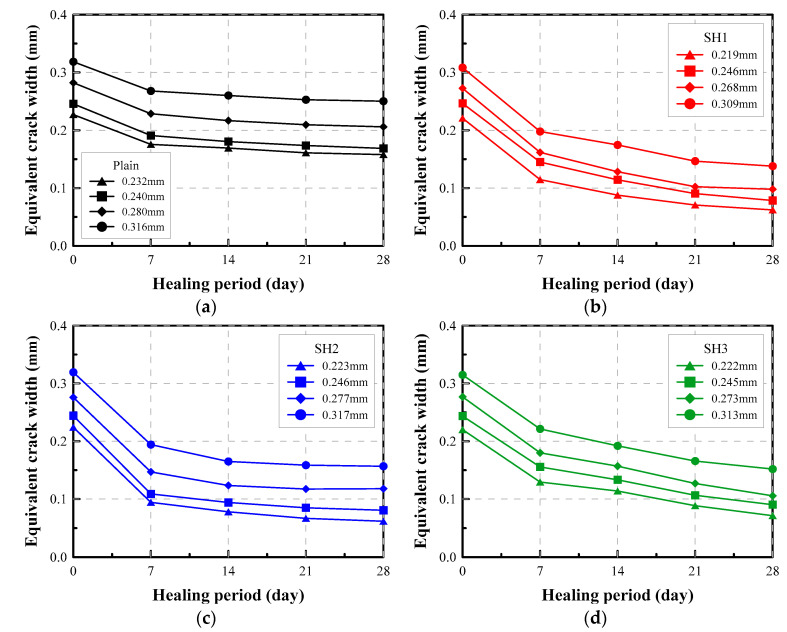
Equivalent crack width of selected specimens: (**a**) Plain, (**b**) SH1, (**c**) SH2, (**d**) SH3.

Figure 10 shows graphically the variation of the equivalent crack widths estimated from the water flow rate with an increasing healing period. It can be seen that the crack widths of several specimens decrease to below 0.1 mm with the aid of the self-healing additives, while the crack of the Plain mixture was not sealed to below 0.17 mm width. 

Table 3 shows the measured crack width and water flow rate of selected specimens before and after healing. The table shows that a crack, of a width of less than 0.25 mm, could be sealed to below 0.1 mm with the aid of the self-healing agents. A typical allowable crack width generally specified in the design code for hydraulic reinforced concrete structures is 0.1 mm. It should be noted that by using the equivalent crack width the self-healing performance could be shown more clearly than when expressed in terms of the flow rate.

**Figure 11:**
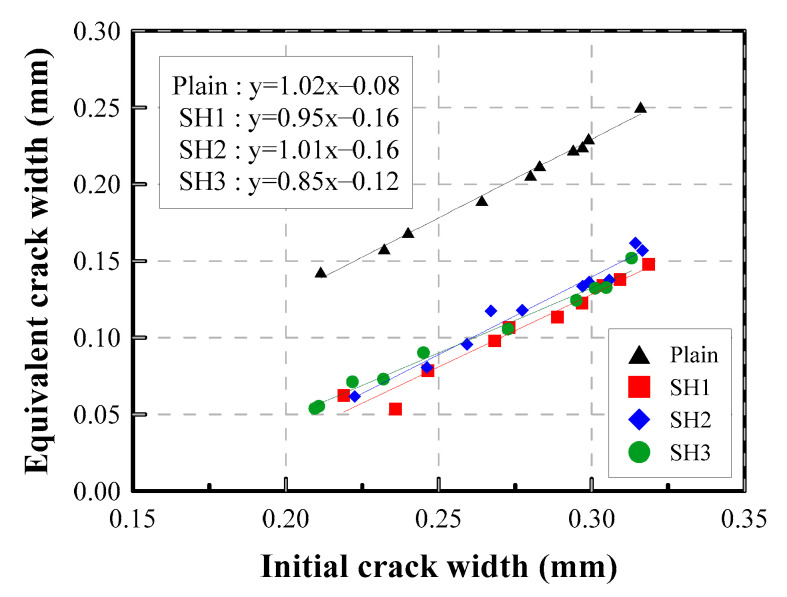
Relationship between the crack width at 28 days and the initial crack width.

The final crack width that can be attained though the healing process is one of the most important factors for engineers when choosing self-healing materials. The relationship between the initial crack width and the crack width after 28 days could be useful information for this purpose, as shown in Figure 11. The *x*-axis represents the initial crack width, and the *y*-axis represents the equivalent crack width after 28 days of healing. The figure shows that these two crack widths are linearly related with above 96% of R-squared. Thus, based on the equations derived, the potential self-healing performance can be predicted accordingly. For the tested mixtures, a 0.26 mm crack can be healed to 0.1 mm by using the SH2 and SH3 mixtures, and a 0.27 mm crack can be reduced to 0.1 mm with the SH1 mixture.

#### 4.3.5. Self-Healing Performance Chart for a Cementitious Material

The test results and the previous studies prove that self-healing is mainly determined not only by the constituents of healing materials, but also by various factors, such as the time at which cracking occurred, the initial crack width, and the healing period. Therefore, if someone wants to evaluate the self-healing performance of a healing material, this could be conducted by a comprehensive analysis of a variety of experimental results performed under different conditions. Therefore, in this study a self-healing performance chart is proposed as a way to readily demonstrate the self-healing performance of healing materials. This chart includes a major index that shows the self-healing performance effectively. The performance chart shows both the evolution of the equivalent crack width (on the *x*-axis) and the healing index (on the *y*-axis) with time. By using this chart, the healing process of a crack of a certain width can be identified for the specific self-healing materials. 

**Figure 12:**
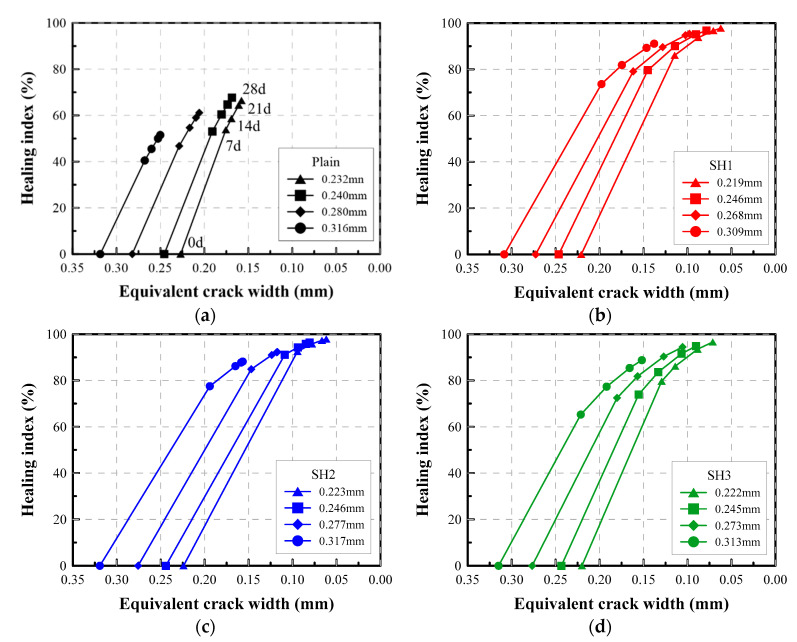
Variation of the crack width and the healing index for each mixture: (**a**) Plain, (**b**) SH1, (**c**) SH2, (**d**) SH3.

Figure 12 is an example of the performance chart for the mixtures tested in this study. This chart allows for a comparison of the major healing parameters at a glance. When comparing the self-healing mixtures with the Plain mixture, the self-healing materials exhibit very large improvements in both the reduction in the crack width and the water flow rate. The Plain mixture cannot reduce the crack width to below 0.15 mm regardless of the initial crack width and more than 30% of the initial flow rate still passes through the crack. However, for self-healing mixtures, the cracks can be sealed below a 0.10 mm width, although the initial crack widths influence the final crack widths attained through the healing process.

For example, an initial crack of 0.219 mm made on an SH1 specimen could be reduced to 0.115, 0.088, 0.071, and 0.062 mm after 7, 14, 21, and 28 days of the healing period, respectively. Accordingly, the healing index increases to 86, 94, 97, and 99%, respectively. Thus, the specific amount to which the crack width can be healed, or the flow rate can be reduced, from the given initial crack width are clearly traced using the proposed chart. If a performance chart is prepared by conducting a series of permeability tests according to the proposed test conditions and methods, the self-healing performance of a specific self-healing material can be efficiently illustrated, allowing engineers to easily expect the self-healing performance of the given material. It should be noted that the experiments were conducted without considering any loading effect that could influence the self-healing processes.

## 5. Conclusions

This study proposes a method and protocol for the evaluation of self-healing performance based on a water permeability test. The performance of three self-healing mixtures have been investigated and compared for the verification of the theory, test method, and the analysis methods.

(1) Three self-healing mixtures incorporating inorganic materials and one Plain mixture were selected for the study. The water/binder ratio and the sand/cement ratio were kept constant for every mixture. Each self-healing mixture had its own characteristics, such as a combination of several constituents, use of clinker, or encapsulation, but they were designed to heal 0.3 mm cracks to a 90% level. The results showed that, as expected, the self-healing performance of these self-healing mixtures were much improved compared to the Plain mixture.
(2) Because many factors related to the permeability test apparatus and method might affect the test results, a detailed setup and method for the water permeability test were proposed as a part of the standardized performance evaluation of the self-healing materials. Based on the proposed method, it was verified that the water flow rate was linearly proportional to the cube of the crack width by using a total of 160 specimens with crack widths ranging from 70 to 320 µm. The crack width can be accurately estimated from the flow rate using a theoretical equation, although the relationship between the crack width and the water flow rate varied with respect to the mixture type.
(3) To evaluate the self-healing performance, several parameters were analyzed and discussed. The water flow rate can be a primary index for the evaluation of self-healing performance. However, the value itself does not produce an intuitive understanding of the condition of the specimens. This study shows that the relative self-healing index is a more rational way to represent the change of permeability than the flow rate itself. In addition, the use of an equivalent crack width could be a reasonable method to directly understand the test results and relate them to crack widths.
(4) It was proven that the initial crack width is linearly related to the healed equivalent crack width or the self-healing index. The equations for these relationships were estimated for each mixture based on the test results. Thus, it was proven that the condition of a crack after healing can be estimated from the initial crack width, or the maximum allowable crack width can be suggested based on the expected healing performance. In addition, a self-healing performance chart was proposed, which shows the variations of the crack width and the healing index during the self-healing process.

## Figures and Tables

**Table 1:** Mix proportions for the Plain and three self-healing mortar mixtures.

Mix Type	Water	Cement	GGBFS	Fine Aggregate	Healing Agent
Plain	0.40	1.00	0	2.00	-
SH1	0.40	1.00	0	1.95	0.05
SH2	0.40	1.00	0	1.92	0.08
SH3	0.40	0.65	0.20	1.80	0.35

**Table 2:** Slump flow and compressive strength test results for mortar mixtures.

Types	Slump Flow (mm)			Compressive Strength (MPa)	
	0 min	30 min	60 min	7 d	28 d
Plain	180	160	150	38.43	49.96
SH1	180	160	145	40.74	53.24
SH2	195	170	140	35.01	46.12
SH3	175	145	125	47.41	54.02

**Table 3:** Water permeability test results for selected crack widths.

Classification		Crack Width [mm]				Water Flow Rate [mL/(mm∙min)]				
		$w_{0}$	${\bar{w}}_{0}$ (Equation (3))	$\left|\frac{w_{0}-{\bar{w}}_{0}}{w_{0}}\right|$ (%)	${\bar{w}}_{28}$ (Equation (4))	$q_{0}$	${\bar{q}}_{0}$ (Equation (2))	$\left|\frac{q_{0}-{\bar{q}}_{0}}{q_{0}}\right|$ (%)	$q_{28}$	$SH_{q}$ (%) (Equation (5))
Plain	1	0.211	0.215	−1.59	0.143	0.61	0.58	4.61	0.18	70.65
	2	0.232	0.227	2.20	0.158	0.72	0.77	−6.89	0.24	66.44
	3	0.240	0.246	−2.37	0.169	0.92	0.86	6.78	0.30	67.70
	4	0.264	0.262	0.66	0.189	1.12	1.14	−2.00	0.42	62.43
	5	0.280	0.282	−0.76	0.206	1.39	1.36	2.24	0.54	61.15
	6	0.283	0.285	−0.64	0.212	1.43	1.40	1.89	0.59	58.74
	7	0.294	0.296	−0.78	0.222	1.61	1.57	2.30	0.68	57.76
	8	0.297	0.296	0.45	0.224	1.60	1.62	−1.35	0.70	56.25
	9	0.299	0.303	−1.30	0.230	1.72	1.65	3.80	0.75	56.40
	10	0.316	0.319	−0.79	0.250	2.00	1.95	2.34	0.97	51.50
SH1	1	0.219	0.221	−0.88	0.062	0.74	0.65	12.88	0.02	97.76
	2	0.236	0.235	0.23	0.053	0.90	0.81	9.92	0.01	98.83
	3	0.246	0.246	−0.02	0.078	1.04	0.93	10.60	0.03	96.78
	4	0.268	0.273	−1.59	0.098	1.40	1.20	14.69	0.06	95.36
	5	0.273	0.274	−0.47	0.107	1.43	1.26	11.81	0.08	94.14
	6	0.289	0.292	−1.03	0.113	1.72	1.49	13.24	0.10	94.13
	7	0.297	0.297	−0.06	0.123	1.81	1.62	10.71	0.13	92.97
	8	0.304	0.308	−1.43	0.134	2.02	1.73	14.27	0.17	91.75
	9	0.309	0.308	0.29	0.138	2.03	1.83	9.78	0.18	91.06
	10	0.319	0.324	−1.69	0.148	2.35	2.00	14.93	0.22	90.49
SH2	1	0.223	0.224	−0.70	0.062	0.73	0.68	6.59	0.02	97.92
	2	0.246	0.244	0.80	0.081	0.95	0.92	2.30	0.03	96.41
	3	0.259	0.257	1.02	0.096	1.10	1.08	1.65	0.06	94.83
	4	0.267	0.271	−1.38	0.117	1.29	1.18	8.46	0.11	91.85
	5	0.277	0.276	0.53	0.118	1.36	1.32	3.08	0.11	92.19
	6	0.297	0.299	−0.50	0.133	1.73	1.62	6.04	0.15	91.08
	7	0.299	0.298	0.56	0.136	1.71	1.66	2.99	0.16	90.41
	8	0.306	0.302	1.12	0.138	1.79	1.77	1.35	0.17	90.57
	9	0.314	0.319	−1.62	0.162	2.12	1.92	9.12	0.27	87.05
	10	0.317	0.319	−0.85	0.157	2.11	1.96	7.02	0.25	88.17
SH3	1	0.209	0.210	−0.11	0.054	0.60	0.57	4.80	0.01	98.31
	2	0.211	0.213	−0.95	0.055	0.62	0.58	7.15	0.01	98.25
	3	0.222	0.220	0.74	0.071	0.69	0.68	2.34	0.02	96.62
	4	0.232	0.232	−0.16	0.073	0.81	0.77	4.93	0.03	96.90
	5	0.245	0.244	0.61	0.090	0.94	0.91	2.72	0.05	94.91
	6	0.273	0.277	−1.51	0.106	1.37	1.25	8.69	0.08	94.45
	7	0.295	0.297	−0.78	0.124	1.70	1.59	6.66	0.12	92.68
	8	0.301	0.306	−1.65	0.132	1.86	1.69	9.05	0.15	91.94
	9	0.305	0.304	0.29	0.133	1.82	1.75	3.63	0.15	91.68
	10	0.313	0.315	−0.59	0.152	2.02	1.90	6.14	0.23	88.78

## Data Availability

Data available on request due to restrictions eg privacy or ethical.
